# Effects of increasing acetate, butyrate, and β-hydroxybutyrate on expression of lipogenic genes in bovine mammary epithelial cell culture

**DOI:** 10.3168/jdsc.2026-1091

**Published:** 2026-08-03

**Authors:** Abiel Haile, Kevin J. Harvatine

**Affiliations:** Department of Animal Science, Penn State University, University Park, PA 16802

## Abstract

Acetate and butyrate are the main lipogenic short-chain fatty acids (SCFA). β-Hydroxybutyrate is synthesized primarily from butyrate and can also serve as a substrate for mammary de novo fatty acid synthesis. Acetate supplementation in vivo increases milk fat yield, but the direct effects of SCFA and BHB on transcriptional regulation of lipid synthesis in mammary epithelial cells remain unclear. This study was designed to specifically evaluate the direct effects of increasing sodium butyrate, sodium acetate, and BHB on lipogenic gene expression in mammary epithelial cells (MAC-T). Cells were treated for 24 h with 0, 4, 8, or 12 m*M* of each, and expression of the *ACSS2*, *ACACA*, *FASN*, *SCD1*, *INSIG1*, and *SREBF1C* genes were analyzed. Butyrate increased lipogenic genes to the greatest extent, with a dramatic increase in *SREBF1c* (>14-fold at 12 m*M*), *FASN*, *ACACA*, and *SCD1*. Sodium acetate had modest effects, increasing *ACSS2* and *SCD1* expression without broad activation of other lipogenic genes. However, BHB increased *FASN* (∼16-fold at 8 m*M*) but had minimal effects on *SREBF1c*, *INSIG1*, or *SCD1*. We found that BHB also increased *PPARG* and *FABP3*, which was not observed with acetate or butyrate. In summary, butyrate strongly increased expression of mammary lipogenic genes, consistent with both its role as a preferred primer for fatty acid synthesis and reported HDAC-inhibitory activity. Acetate and BHB exerted more limited, pathway-specific responses, underscoring potentially distinct mechanisms of SCFA-mediated regulation of expression of lipid metabolism enzymes in the mammary gland. Future work will be needed to determine the functional roles on the rate of lipid synthesis.

## Graphical Abstract

**Figure:**
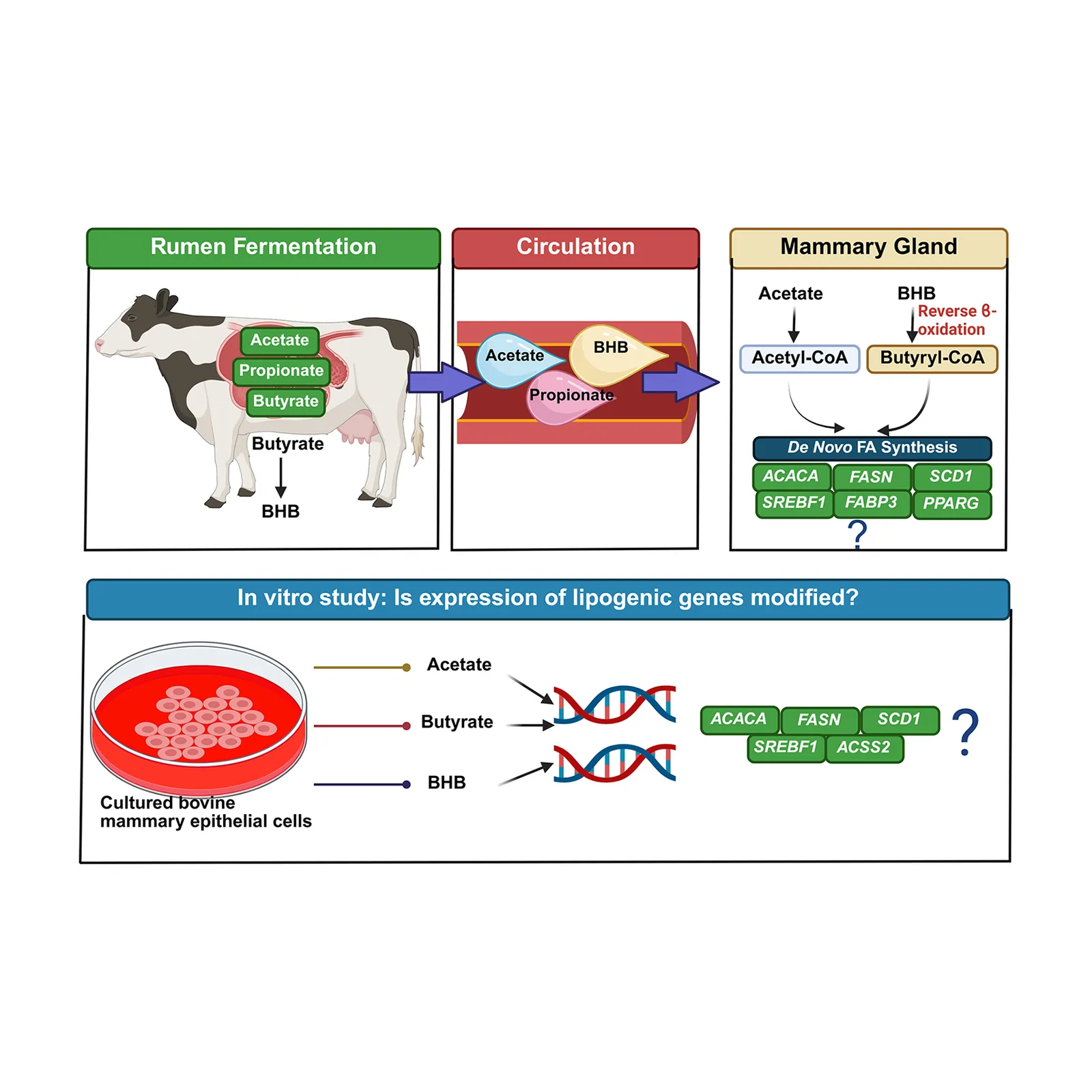

Milk fat synthesis in the bovine mammary gland is a tightly regulated metabolic process influenced by both substrate availability and major regulation at the level of gene expression (Harvatine et al., 2009). Biochemically, acetate, butyrate, and ketone bodies can support de novo fatty acid (**FA**) synthesis, can contribute to cellular energy balance, and may transcriptionally regulate lipid synthesis enzymes. Acetate is readily available to the mammary gland, whereas butyrate availability to the mammary gland is limited by its high rate of oxidation and conversion to BHB. In vivo, acetate supplementation increases both plasma acetate and BHB and has consistently increased milk fat yield in dairy cows, mostly through an increase in 16-carbon FA (Urrutia and Harvatine, 2017), although the molecular mechanism is not clear.

Acetate is the most abundant short-chain FA (**SCFA**) in the rumen, typically accounting for over 60% of the total SCFA in the rumen. It serves as the main carbon source for de novo FA synthesis in the mammary gland and provides half of the reducing equivalents for fat synthesis (Harvatine et al., 2009). Additionally, acetate is a preferred oxidative fuel in ruminants, as classic studies demonstrated that acetate is preferentially oxidized over glucose and long-chain FA in bovine tissues, including the mammary epithelium (Annison et al., 1967).

Butyrate is the third most abundant SCFA and is ∼10% of total SCFA in the rumen in contemporary diets fed to dairy cows. In the mammary gland, butyrate contributes acetyl-CoA and serves as a preferred primer for de novo FA synthesis via its conversion to butyryl-CoA, which more efficiently initiates chain elongation than acetyl-CoA (Palmquist et al., 1969; Lin and Kumar, 1971). In addition to its metabolic role, butyrate has been demonstrated to function as a potent HDAC inhibitor in other models, potentially promoting histone acetylation and transcriptional activation. Several studies have demonstrated that butyrate enhances the expression of lipogenic genes, such as *SREBF1C*, *FASN*, and *SCD1*, likely through both metabolic and epigenetic mechanisms (Sun et al., 2016; Chriett et al., 2019).

Butyrate is produced in large quantities during ruminal fermentation but is extensively converted to BHB during absorption across the rumen epithelium and in the liver, resulting in minimal butyrate in peripheral circulation (Kristensen, 2005). Arterial blood concentrations of butyrate in well-fed, post-peak lactating cows are typically very low, averaging ∼0.01 to 0.03 mmol/L, or ∼0.1 to 0.3 mg/dL (Benson et al., 2001; Kristensen, 2005). In contrast, BHB levels in the bloodstream are much higher, commonly ranging from 0.5 to 0.8 mmol/L (5 to 8 mg/dL) in healthy mid- to late-lactation cows in positive energy balance (Izumi et al., 2019; Melendez et al., 2021). Although butyrate acts as a local substrate and signaling molecule in the gut, its high conversion to BHB limits its availability to the mammary gland. However, in mammary epithelial cells, BHB can be metabolized to acetoacetate and then to butyryl-CoA via reverse β-oxidation (Lin and Kumar, 1972); thus it has the potential to support de novo lipogenesis. Although BHB has been shown to increase expression of genes such as *FASN* and *ACACA* under moderate concentrations in vitro and in vivo (Ali et al., 2021; Zhang et al., 2023), the epigenetic effects are weaker than those of butyrate. Specifically, BHB induces limited global histone acetylation and functions as a comparatively weak HDAC inhibitor (Chriett et al., 2019).

Despite the increasing recognition of acetate, butyrate, and BHB in lipogenic regulation, many studies have assessed them in isolation or without differentiating the regulatory effects of butyrate from those of its major circulating derivative, BHB. This distinction is crucial, given their divergent cellular uptake, metabolism, and transcriptional consequences. Although butyrate acts as both a substrate and a regulator, the effects of BHB may be constrained by its reliance on reverse β-oxidation and its modest epigenetic potency.

Therefore, the objective of the current study was to determine the effects of sodium acetate, sodium butyrate, and sodium BHB on the expression of key de novo lipogenic genes in MAC-T bovine mammary epithelial cells. This study focuses on shorter-term direct effects on transcriptional regulation of lipogenic genes as a potential mechanism rather than demonstration of functional endpoints (e.g., lipid synthesis or protein abundance) that may be better investigated in vivo. We hypothesized that acetate would stimulate specific lipogenic genes, butyrate would activate a broader gene network, and BHB would have more limited effects. Understanding how these SCFA and ketone bodies differentially regulate lipogenic gene expression is expected to provide mechanistic insight into milk fat synthesis and guide future nutritional strategies aimed at enhancing lactation performance.

Immortalized bovine mammary epithelial cells (MAC-T; provided by Y. Boiscliar, Cornell University, Ithaca, NY; passages 14 and 15 were cultured in Dulbecco's Modified Eagle Medium [**DMEM**]; Thermo Fisher Scientific, Waltham, MA) supplemented with 5% fetal bovine serum (Gibco, Thermo Fisher Scientific), 10 µg/mL insulin (Sigma-Aldrich, St. Louis, MO), and 1% penicillin-streptomycin (Thermo Fisher Scientific). Cells were incubated at 37°C in a humidified atmosphere containing 5% CO_2_ and passed upon reaching 70% to 80% confluence.

For experiments, cells were seeded into 6-well plates (1.0 × 10^6^ cells/well), grown to ∼80% confluence, and then serum-starved for 18 h in DMEM before application of treatments for 24 h as will be described. Serum starvation was used to reduce background hormonal and nutrient signaling before treatment. Cells were treated for 24 h with 0, 4, 8, and 12 m*M* sodium acetate (≥99%, Sigma-Aldrich, catalog no. S2889), sodium butyrate (≥98%, Sigma-Aldrich, catalog no. 303410), or sodium BHB (NaBHB; ≥98%, Sigma-Aldrich, catalog no. 54965), in DMEM with no insulin or serum. Insulin and serum were omitted during the treatment period to minimize confounding signaling and the potential that expression of the lipogenic enzymes would be fully stimulated and limit the ability to see an increase from the treatments. The pH of the medium was measured after addition of acetate, butyrate, or BHB at the tested concentrations and did not differ from control wells (**CON**), indicating adequate buffering from the medium.

Following 24 h of treatment, cells were washed twice with cold PBS and lysed with a phenol-guanidine salt solution. Total RNA was extracted using the EZNA Total RNA Kit II (Omega Bio-tek, Norcross, GA) with on-column DNase digestion according to the manufacturer's instructions. Concentration and purity of RNA were assessed using a NanoDrop spectrophotometer (Thermo Fisher Scientific, Waltham, MA). All RNA samples exhibited absorbance ratios at 260 and 280 nm between 1.90 and 2.10. Two micrograms of total RNA was reverse-transcribed using the High-Capacity cDNA Reverse Transcription Kit (Applied Biosystems, Foster City, CA) in a 20-µL reaction volume using random primers. The resulting cDNA was stored at −80°C until further analysis.

Gene expression was analyzed by quantitative real-time reverse-transcription PCR (**qPCR**) using SYBR Green PCR Master Mix (Quanta; Bio-Rad Laboratories, Hercules, CA) in a 384-well Applied Biosystems 7900HT Real-Time PCR System (Thermo Fisher Scientific, Foster City, CA). Each qPCR reaction (10 µL total volume) contained 400 n*M* of each primer (Supplemental Table S1, see Notes) and 10 ng of cDNA. The thermal cycling conditions were as follows: initial denaturation at 95°C for 3 min, followed by 40 cycles of 95°C for 10 s and 60°C for 30 s. Melt curve analysis was performed to confirm a single amplicon. Quantification was performed based on the relative quantification method using standard curves generated from serial dilutions of pooled cDNA. Target genes included lipogenic markers (*SREBF1c*, *INSIG1*, *ACACA*, *FASN*, *ACSS2*, and *SCD1*) and additional lipid-related genes (e.g., *PPARG*, *FABP3*, *THRSP* as applicable), with *ACTB*, *B2M*, and *18S* rRNA used as reference genes. A normalization factor (**NF**) was calculated for each sample as the geometric mean of the quantities of 3 reference genes (*ACTB*, *B2M*, and *18S* rRNA), scaled to the geometric mean across all samples. Target gene quantities were normalized by dividing the target quantity by the sample-specific NF.

Data were analyzed using the General Linear Model (GLM) procedure in SAS 9.4 (SAS Institute Inc., Cary, NC), with dose (0, 4, 8, 12 m*M*) as the fixed effect within each compound. Mean comparisons were conducted using a protected LSD test when the effect of dose was significant at *P* < 0.05. Data are plotted relative to CON (0 m*M*) on a log-scaled axis to more accurately represent the magnitude of decreases in expression. Lower error bars are omitted when their bounds fall below the minimum y-axis value.

Sodium acetate elicited a dose-dependent increase in 2 lipogenic transcripts, *ACSS2* and *SCD1* (*P* = 0.04), but had no effect on *FASN*, *ACACA*, or *INSIG1* (Figure 1). Expression of ACSS2 was increased ∼1.6-fold at 8 m*M* acetate, but 4 and 12 m*M* were not different from CON. Expression of *SCD1* was numerically increased at 4 m*M* and increased ∼2-fold at 8 and 12 m*M* acetate compared with CON (*P* = 0.02). Expression of *SREBF1c* was numerically increased at 4 m*M* sodium acetate compared with CON and was decreased by 12 m*M* compared with 4 m*M* but was not different from the CON (*P* = 0.03). No cytotoxicity was visually observed in acetate-treated cells at any concentration.

**Figure 1:**
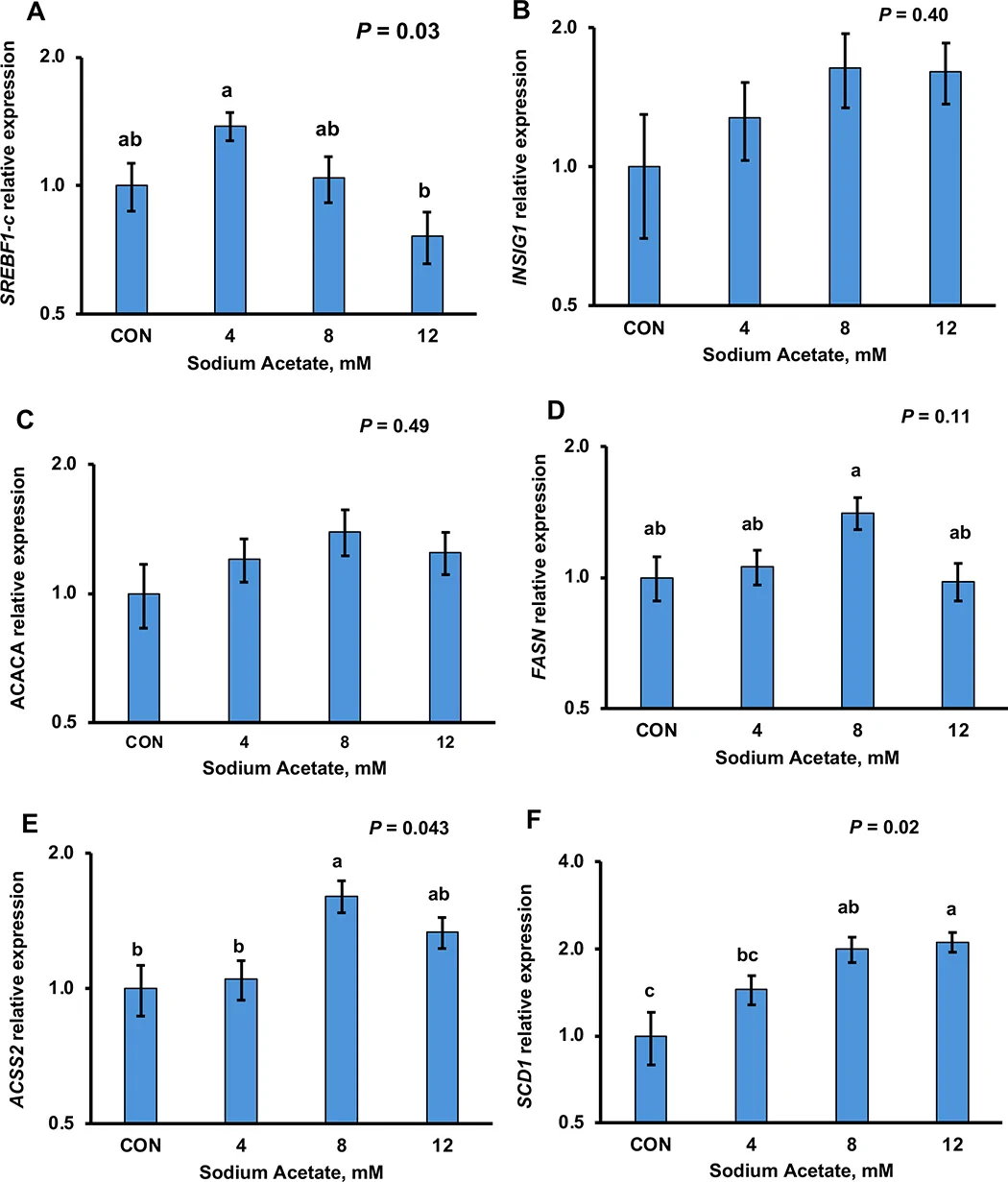
Effects of sodium acetate on expression of lipogenic genes in MAC-T bovine mammary epithelial cells. Cells were treated for 24 h with 0 (CON), 4, 8, or 12 m*M* sodium acetate, and mRNA abundances of (A) *SREBF1c*, (B) *INSIG1*, (C) *ACACA*, (D) *FASN*, (E) *ACSS2*, and (F) *SCD1* were quantified by qPCR and normalized to the geometric mean of 3 reference genes (*ACTB*, *B2M*, and *18S rRNA*). Data are presented as fold change relative to the control group (mean ± SEM; n = 3 biological replicates) and plotted on a log axis. Means that share a letter do not differ (*P* > 0.05).

Sodium butyrate markedly upregulated all quantified lipogenic genes (Figure 2). Expression of *ACSS2* was increased 3.9-fold at 4 m*M* and progressively increased over 12-fold at 12 m*M* compared with CON (*P* < 0.0001). Expressions of *FASN* and *ACACA* were also increased progressively up to 8 m*M*, but were not increased between 8 and 12 m*M* butyrate. Specifically, FASN was increased up to ∼3.3-fold, and ACACA was increased up to ∼3.0-fold. Expression of *SCD1* was nearly equally increased ∼3.5-fold by all doses of butyrate.

**Figure 2:**
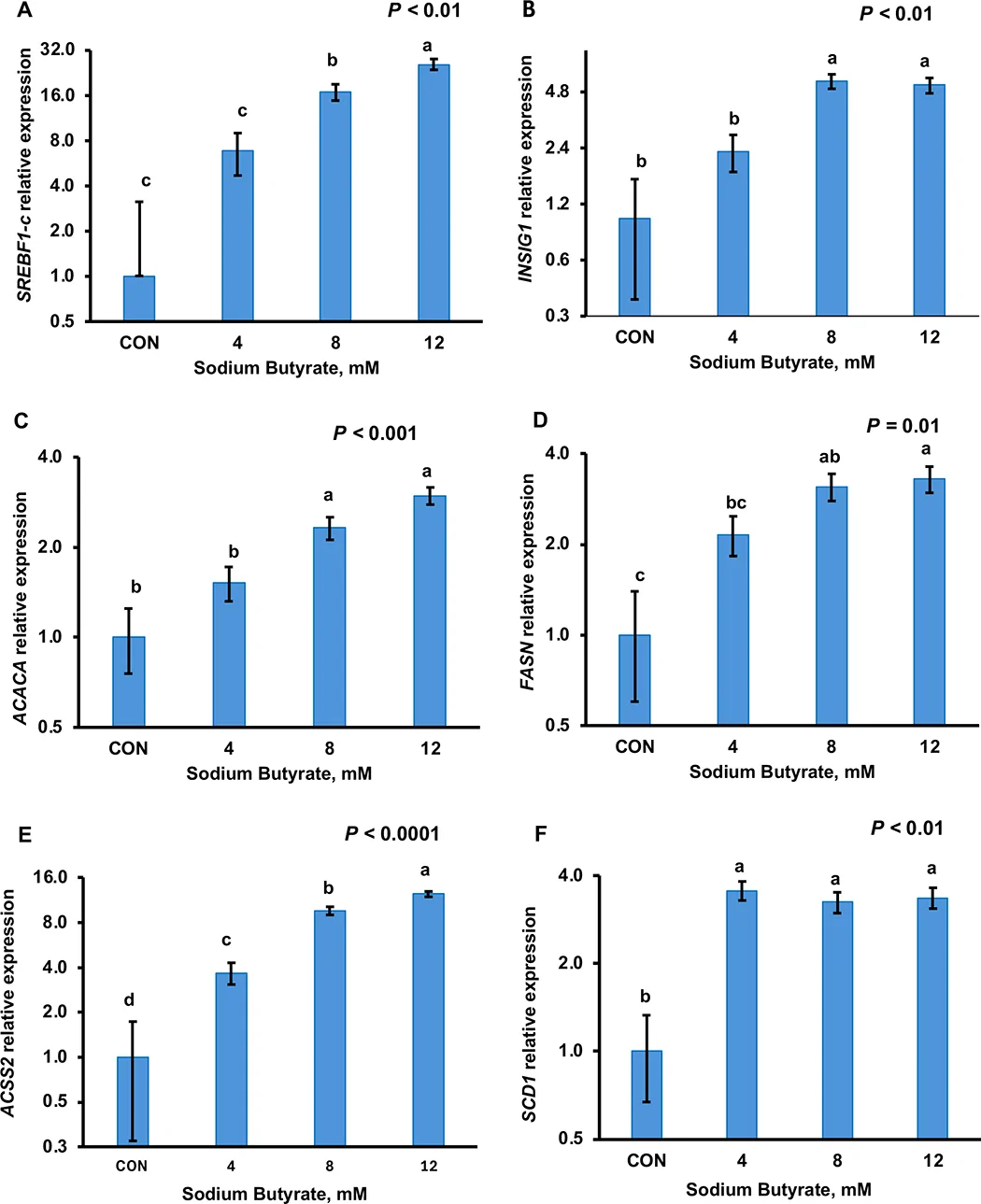
Effects of sodium butyrate on expression of lipogenic genes in MAC-T bovine mammary epithelial cells. Cells were treated for 24 h with 0 (CON), 4, 8, or 12 m*M* sodium butyrate, and mRNA abundances of (A) *SREBF1c*, (B) *INSIG1*, (C) *ACACA*, (D) *FASN*, (E) *ACSS2*, and (F) *SCD1* were measured and normalized to internal controls as described in Figure 1. Data are shown as fold change relative to control (mean ± SEM; n = 3) and plotted on a log axis. Means that share a letter do not differ (*P* > 0.05). Lower error bars are not shown when their bounds fall below the lowest value of the y-axis.

Expressions of *SREBF1c* and *INSIG1* were also increased by butyrate (*P* < 0.01; Figure 2). At 4 m*M* butyrate, *SREBF1c* and *INSIG1* were not different from control. However, *SREBF1c* expression was dramatically increased 16.8-fold at 8 m*M* and 25-fold at 12 m*M* sodium butyrate compared with control. Expression of *INSIG1* was increased ∼5.5-fold at 8 m*M* and 6.2-fold at 12 m*M*, although no difference occurred between 8 and 12 m*M* sodium butyrate.

The effects of sodium BHB on lipogenic gene expression were less consistent (Figure 3). Expression of *ACSS2* and *SCD1* were not altered by BHB (*P* = 0.32 and 0.21). Expression of *FASN* was the most responsive to sodium BHB (*P* = 0.01) with an increase of ∼8-fold at 4 m*M* and ∼16-fold at 8 m*M*, with no additional increase at 12 m*M*. Although *ACACA* expression was affected by BHB (*P* = 0.002), it was decreased at 4 and 12 m*M* and was not different from control at the 8 m*M* dose. Neither *INSIG1* (data not shown) nor *SREBF1c* expression was affected by BHB treatment (*P* > 0.30). Expression of *THRSP* tended to be increased (*P* = 0.06), although this effect was not observed with other treatments (data not shown). Notably, sodium BHB increased expression of *PPARG* (*P* < 0.01) and *FABP3* (*P* < 0.01), whereas acetate and butyrate did not affect these genes. These responses were specific to BHB treatment and were not observed for the other SCFA.

**Figure 3:**
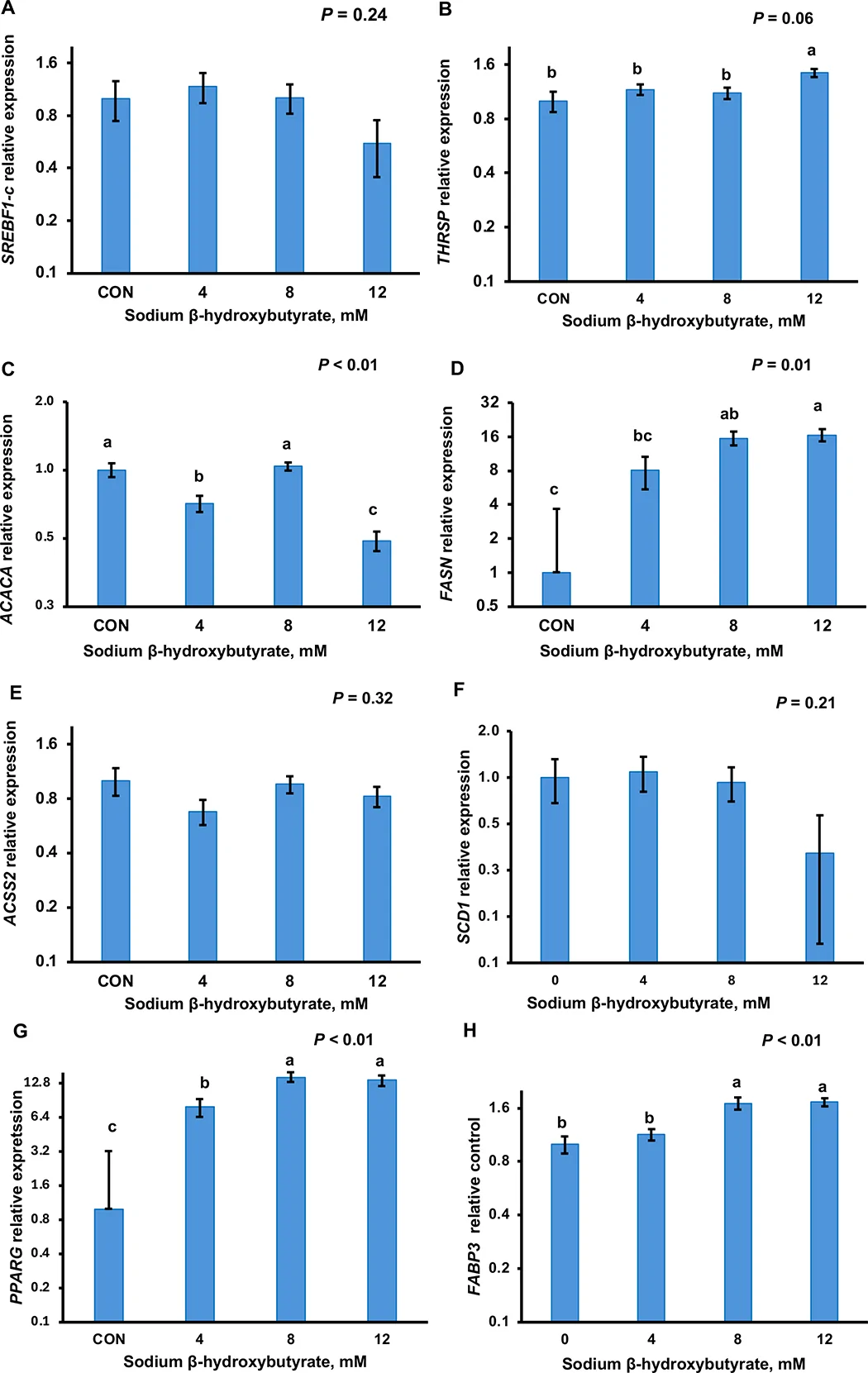
Effects of sodium BHB on expression of lipogenic genes in MAC-T bovine mammary epithelial cells. Cells were treated for 24 h with 0 (control), 4, 8, or 12 m*M* sodium BHB, and mRNA abundances of (A) *SREBF1c*, (B) *THRSP*, (C) *ACACA*, (D) *FASN*, (E) *ACSS2*, (F) *SCD1*, (G) *PPARG*, and (H) *FABP3* were determined by qPCR and normalized to the geometric mean of reference genes. Data are shown as fold change relative to control (mean ± SEM; n = 3) and plotted on a log axis. Means that share a letter do not differ (*P* > 0.05). Lower error bars are not shown when their bounds fall below the lowest value of the y-axis.

In the current study, sodium acetate, sodium butyrate, and sodium BHB differentially regulated the expression of lipogenic genes in bovine mammary epithelial cells in culture. Both butyrate and acetate upregulated key enzymes and transcriptional regulators involved in de novo FA synthesis, but sodium butyrate exerted a much more robust response. However, BHB exhibited a more selective and modest effect. A consistent trend across all treatments was that maximal gene expression responses were typically observed at moderate concentrations (4–8 m*M*), with diminished or plateaued effects at higher doses. This reinforces the importance of SCFA concentration in determining transcriptional outcomes. It is important to recognize that these doses are much higher than plasma concentrations and also likely higher than cellular concentrations, as is common in for in vitro systems. The responses show a dynamic dose-dependent response, but future work should explore doses lower than 4 m*M* and validate responses in vivo.

Sodium acetate increased expression of *ACSS2* and *SCD1* in a dose-dependent manner, while having no effect on *FASN*, *ACACA*, or *INSIG1*. The upregulation of *ACSS2*, the enzyme responsible for converting acetate to acetyl-CoA, may underscore acetate's role as a direct carbon source for FA synthesis. Although *SREBF1c* expression was only modestly affected, the elevated expression of downstream enzymes supports the notion that acetate may enhance lipogenesis through post-transcriptional or signaling mechanisms rather than through strong transcriptional activation of *SREBF1c*. These findings are consistent with previous in vivo studies showing that acetate infusion enhances milk fat yield (Urrutia and Harvatine, 2017) and with evidence that acetate is preferentially oxidized over glucose in the mammary gland (Annison et al., 1967). Although our results did not observe upregulation of *FASN* or *ACACA*, prior studies have demonstrated acetate's ability to stimulate these genes under different experimental conditions. For instance, Lin et al. (2022) reported that sodium acetate enhanced *FASN* and *ACACA* expression in bovine mammary epithelial cells via activation of the mTORC1 pathway, and Zhao et al. (2021) reported that acetate increased lipogenesis through an mTOR/eIF4E-dependent mechanism. Likewise, Qi et al. (2023) found that acetate activated GPR41, GPR43 signaling, and mTORC1 signaling in HC11 mammary epithelial cells, promoting lipid droplet formation. Additionally, the response was inhibited by GPR21 and GPR43 knockdown. In contrast, the lack of *FASN* and *ACACA* response in our study may reflect dose- or context-specific signaling thresholds. Ali et al. (2021) also reported that acetate increased triglyceride content and upregulated *SREBF1c* in combination with BHB, suggesting synergistic effects not replicated with acetate alone. These discrepancies highlight the complexity of acetate signaling and the importance of cellular context in interpreting its effects.

However, consistent with our results, Sun et al. (2016) reported that sodium acetate modestly increased *SCD1* expression in goat mammary epithelial cells, despite having no effect on triglyceride accumulation or lipid droplet formation. This parallels our observation that acetate selectively upregulated *SCD1* and *ACSS2*, both of which are linked to de novo FA synthesis, as *ACSS2* provides acetyl-CoA from acetate and *SCD1* contributes to desaturation of newly synthesized saturated FA. In contrast, sodium butyrate strongly induced the de novo lipogenic program in both studies, markedly enhancing expression of key regulators, such as *SREBF1c*, *FASN*, *ACACA*, and *SCD1* (Harvatine and Bauman, 2006). The conservation of these effects across caprine and bovine mammary cell models supports the broader relevance of butyrate in regulating milk fat synthesis at the molecular level.

Sodium butyrate elicited the most pronounced transcriptional activation, upregulating all measured lipogenic genes and robustly increasing expression of *SREBF1*c, indicating robust activation of the lipogenic program. Butyrate is known to serve as a preferred primer for de novo FA synthesis through its conversion to butyryl-CoA, which is more efficient than acetyl-CoA at initiating FA elongation in the mammary gland (Lin and Kumar, 1971). Additionally, butyrate exerts potent epigenetic effects via inhibition of HDAC. Chriett et al. (2019) confirmed that butyrate is a more effective HDAC inhibitor than BHB, enhancing histone acetylation and transcriptional activation in multiple cell types. In our experiment, butyrate increased expression of *SREBF1*c and its downstream targets, consistent with HDAC inhibition-mediated chromatin remodeling. This is supported by Shin et al. (2021), who showed that HDAC1 suppresses *SREBF1c* in sebocytes, and that HDAC1 knockdown increases *SREBF1c* expression and lipogenesis. Although chromatin remodeling was not directly measured in the current work, our results align with this mechanism and open the possible dual role of butyrate as a substrate and a chromatin-modifying regulator.

There have been limited investigations of butyrate supplementation in vivo, and these are difficult to interpret due to differences in experimental power and doses. It is well established that ruminal butyrate infusion has limited effect on plasma butyrate. For example, Herrick et al. (2018) reported that 717 and 1,434 g/d of butyrate increased plasma butyrate from 0.08 to 0.11 m*M*. Huhtanen et al. (1993) reported that rumen infusion of 400 and 600 g/d of butyrate increased plasma butyrate from 0.012 and 0.026 m*M*. Milk fat responses also vary. For example, Herrick did not observe a milk fat response, but this may be due to negative effects on intake and milk yield. Huhtanen et al. (1993) reported that 400 and 600 g/d of butyrate increased milk fat yield by 51 and 74 g/d. Feeding butyrate at 1.1% of diet DM increased milk and milk fat yield more than 80 g/d in Izumi et al. (2019). Zhang et al. (2023) fed 100, 200, and 300 g/d of sodium butyrate and observed a linear increase in milk fat of up to 260 g/d. More recently, Guo et al. (2021) fed tributyrin at 0.1% of diet DM to heat-stressed Holstein cows and reported a ∼20% increase in milk fat concentration, along with reductions in markers of inflammation and oxidative stress. In contrast, a study from our laboratory found that supplementing 600 g/d of calcium butyrate had no effect on milk fat yield under non–heat stress conditions (Urrutia et al., 2019). The very small increases in plasma butyrate relative to the doses used in the current study question whether increased milk fat is due to increase butyrate absorption, and responses may be due to associative effects in the rumen or more modest effects of BHB, which is increased to a greater extent.

In the current cell culture experiments, sodium BHB exhibited a more nuanced and gene-specific effect. Expression of FASN was robustly increased by BHB, but the reason for the lack of coordination between *FASN* and *ACACA* is not clear. Notably, BHB did not alter *ACSS2* or *SCD1* and had no meaningful effect on *SREBF1c* or *INSIG1*. In contrast, sodium BHB increased *PPARG* and *FABP3*, and these genes were not responsive to acetate or butyrate, indicating a BHB-specific transcriptional response distinct from coordinated activation of the core de novo lipogenic gene network. This pattern is consistent with regulation that is not accompanied by changes in *SREBF1c* mRNA abundance. Physiologically, the mammary gland is exposed to more BHB than to butyrate, as most ruminal butyrate is converted to BHB in the rumen wall and liver (Kristensen, 2005). Once inside mammary epithelial cells, BHB can be converted to acetoacetate and subsequently to butyryl-CoA via reverse β-oxidation and butyryl-CoA is an efficient primer for FA synthesis as discussed earlier. However, this pathway requires multiple enzymatic conversions, which may limit the efficiency of BHB utilization compared with direct butyrate uptake. Additionally, although BHB can induce histone β-hydroxybutyrylation, it is a relatively weak HDAC inhibitor (Chriett et al., 2019), which may explain its limited effect on transcriptional activation in our study.

The distinct gene expression differences between butyrate and BHB may be attributable to both their metabolic conversion routes and epigenetic influence. Butyrate may directly function as a potent HDAC inhibitor, inducing lipogenic gene expression through transcriptional activation. Expression of lipogenic enzymes is highly regulated by SREBF1, but signaling through other factors should also be investigated. In contrast, BHB must first undergo metabolic transformation before contributing to butyryl-CoA pools and lacks comparable epigenetic potency. Additionally, the differences observed may reflect cell-specific expression of enzymes responsible for BHB metabolism or threshold effects required to activate key transcription factors such as *SREBF1c*.

Overall, our findings emphasize the distinct and concentration-dependent roles of sodium acetate, butyrate, and BHB in regulating lipogenic gene expression in the bovine mammary cells. Acetate primarily supported lipogenesis by upregulating *ACSS2* and *SCD1* but induced limited transcriptional activation of master regulators. Butyrate robustly enhanced expression of *SREBF1c* and downstream targets, likely via HDAC inhibition and its role as a lipogenic primer. Finally, BHB supported selective gene activation but appears limited by its metabolic conversion and weaker epigenetic influence. Because mammary lipogenesis has substantial regulation at the level of gene expression, these changes would be expected to drive increases in milk fat yield. Treatments with broader effects across multiple enzymes would be expected to have a larger influence than treatments that increased a smaller number of genes depending on which steps are limiting.

Key experimental limitations are important to recognize and help prioritize future work. First, our hypothesis focused on effects on transcriptional regulation, as this has been shown to be a key regulated step of milk fat synthesis, but future work should measure effects on protein concentration, enzyme activity, and lipogenic capacity. Additionally, we selected a serum- and insulin-free condition to provide a lower rate of expression of lipogenic enzymes, which would allow the potential for treatments to increase their expression. Replication across other conditions in vitro and in vivo will provide additional insights into interactions with other regulatory systems. Cell culture models have numerous limitations, but it is important to recognize that in vitro systems do allow testing of the direct effect on mammary cells, whereas in vivo responses are confounded by endocrine responses and metabolic effects in nonmammary tissues. Overall, the insights from the current work enhance our understanding of the potential molecular mechanisms by which rumen-derived metabolites regulate mammary lipid metabolism and may inform nutritional strategies aimed at optimizing milk fat synthesis in lactating dairy cows.

## References

[bib1] Ali I., Li C., Li L., Kuang M., Shafiq M., Wang Y., Yang M., Wang G. (2021). Effect of acetate, β-hydroxybutyrate and their interaction on lipogenic gene expression, triglyceride contents and lipid droplet formation in dairy cow mammary epithelial cells. In Vitro Cell. Dev. Biol. Anim..

[bib2] Annison E.F., Linzell J.L., Fazakerley S., Nichols B.W. (1967). The oxidation and utilization of palmitate, stearate, oleate and acetate by the mammary gland of the fed goat in relation to their overall metabolism, and the role of plasma phospholipids and neutral lipids in milk-fat synthesis. Biochem. J..

[bib3] Benson J.A., Reynolds C.K., Humphries D.J., Rutter S.M., Beever D.E. (2001). Effects of abomasal infusion of long-chain fatty acids on intake, feeding behavior and milk production in dairy cows. J. Dairy Sci..

[bib4] Chriett S., Dąbek A., Wojtala M., Vidal H., Balcerczyk A., Pirola L. (2019). Prominent action of butyrate over β-hydroxybutyrate as histone deacetylase inhibitor, transcriptional modulator and anti-inflammatory molecule. Sci. Rep..

[bib5] Guo W., Liu J., Yang Y., Ma H., Gong Q., Kan X., Ran X., Cao Y., Wang J., Fu S., Hu G. (2021). Rumen-bypassed tributyrin alleviates heat stress by reducing the inflammatory responses of immune cells. Poult. Sci..

[bib6] Harvatine K.J., Bauman D.E. (2006). SREBP1 and thyroid hormone responsive spot 14 (S14) are involved in the regulation of bovine mammary lipid synthesis during diet-induced milk fat depression and treatment with CLA. J. Nutr..

[bib7] Harvatine K.J., Boisclair Y.R., Bauman D.E. (2009). Recent advances in the regulation of milk fat synthesis. Animal.

[bib8] Herrick K.J., Hippen A.R., Kalscheur K.F., Schingoethe D.J., Ranathunga S.D., Anderson J.L., Moreland S.C., van Eys J.E. (2018). Infusion of butyrate affects plasma glucose, butyrate, and beta-hydroxybutyrate but not plasma insulin in lactating dairy cows. J. Dairy Sci..

[bib9] Huhtanen P., Miettinen H., Ylinen M. (1993). Effect of increasing ruminal butyrate on milk yield and blood constituents in dairy cows fed a grass silage-based diet. J. Dairy Sci..

[bib10] Izumi K., Fukumori R., Oikawa S., Oba M. (2019). Short communication: Effects of butyrate supplementation on the productivity of lactating dairy cows fed diets differing in starch content. J. Dairy Sci..

[bib11] Kristensen N.B. (2005). Splanchnic metabolism of volatile fatty acids in the dairy cow. Anim. Sci..

[bib12] Lin C.Y., Kumar S. (1971). Primer specificity of mammary fatty acid synthetase and the role of the soluble β-oxidative enzymes. J. Biol. Chem..

[bib13] Lin C.Y., Kumar S. (1972). Pathway for the synthesis of fatty acids in mammalian tissues. J. Biol. Chem..

[bib14] Lin M., Jiang M., Yang T., Tan D., Hu G., Zhao G., Zhan K. (2022). Acetate-induced milk fat synthesis is associated with activation of the mTOR signaling pathway in bovine mammary epithelial cells. Animals (Basel).

[bib15] Melendez P., Bartolomé J., Roeschmann C., Soto B., Arevalo A., Möller J., Coarsey M. (2021). The association of prepartum urine pH, plasma total calcium concentration at calving and postpartum diseases in Holstein dairy cattle. Animal.

[bib16] Palmquist D.L., Davis C.L., Brown R.E., Sachan D.S. (1969). Availability and metabolism of various substrates in ruminants. V. Entry rate into the body and incorporation into milk gat of d(−)β-hydroxybutyrate. J. Dairy Sci..

[bib17] Qi Y., Zheng T., Liu X., Yang S., Li Q., Shao J., Zeng X., Guan W., Zhang S. (2023). Sodium acetate regulates milk fat synthesis through the activation of GPR41/GPR43 signaling pathway. Front. Nutr..

[bib18] Shin H.S., Lee Y., Shin M.H., Cho S.I., Zouboulis C.C., Kim M.K., Lee D.H., Chung J.H. (2021). Histone deacetylase 1 reduces lipogenesis by suppressing SREBP1 transcription in human sebocyte cell line SZ95. Int. J. Mol. Sci..

[bib19] Sun Y., Luo J., Zhu J., Shi H., Li J., Qiu S., Wang P., Loor J.J. (2016). Effect of short-chain fatty acids on triacylglycerol accumulation, lipid droplet formation and lipogenic gene expression in goat mammary epithelial cells. Anim. Sci. J..

[bib20] Urrutia N., Bomberger R., Matamoros C., Harvatine K. (2019). Effect of dietary supplementation of sodium acetate and calcium butyrate on milk fat synthesis in lactating dairy cows. J. Dairy Sci..

[bib21] Urrutia N.L., Harvatine K.J. (2017). Acetate dose-dependently stimulates milk fat synthesis in lactating dairy cows. J. Nutr..

[bib22] Zhang J., Bu L., Liu Y., Huo W., Xia C., Pei C., Liu Q. (2023). Dietary supplementation of sodium butyrate enhances lactation performance by promoting nutrient digestion and mammary gland development in dairy cows. Anim. Nutr..

[bib23] Zhao Y., Guo X., Yan S., Shi B., Sheng R. (2021). Acetate regulates milk fat synthesis through the mammalian target of rapamycin/eukaryotic initiation factor 4E signaling pathway in bovine mammary epithelial cells. J. Dairy Sci..

